# The impacts of allopolyploidization on Methyl-CpG-Binding Domain (*MBD*) gene family in *Brassica napus*

**DOI:** 10.1186/s12870-022-03485-0

**Published:** 2022-03-07

**Authors:** Yafang Xiao, Mengdi Li, Jianbo Wang

**Affiliations:** 1 State Key Laboratory of Hybrid Rice, College of Life Sciences, Wuhan University, Wuhan, 430072 China; 2 Key Laboratory of Resource Biology and Biotechnology in Western China, Ministry of Education, College of Life Sciences, Northwest University, Xi’an, 710069 China

**Keywords:** *MBD* gene family, Allopolyploidization, *Brassica napus*, Expression pattern, Gene structure

## Abstract

**Background:**

Polyploidization promotes species formation and is widespread in angiosperms. Genome changes dramatically bring opportunities and challenges to plants after polyploidy. Methyl-CpG-Binding Domain (MBD) proteins can recognize and bind to methylation sites and they play an important role in the physiological process related to methylation in animals and plants. However, research on the influence of the allopolyploidization process on the *MBD* gene family is still lacking, so it is necessary to conduct a comprehensive analysis.

**Results:**

In this study, twenty-two, ten and eleven *MBD* genes were identified in the genome of allotetraploid *B. napus* and its diploid ancestors, *B. rapa* and *B. oleracea*, respectively. Based on the clades of the *MBD* gene in Arabidopsis, rice and maize, we divided the new phylogenetic tree into 8 clades. Among them, the true *MBD* genes in *Brassica* existed in only 5 clades. Clade IV and Clade VI were unique in term of *MBD* genes in dicotyledons. Ka/Ks calculations showed that *MBD* genes underwent purifying selection in *Brassica* and may retain genes through sequence or functional differentiation early in evolution. In the process of allopolyploidization, the number of *MBD* gene introns increased, and the protein motifs changed. The MBD proteins had their own special motifs in each clade, and the MBD domains were only conserved in their clades. At the same time, the *MBD* genes were expressed in flower, leaf, silique, and stem tissues, and the expression levels of the different genes were significantly different, while the tissue specificity was not obvious. The allopolyploidization process may increase the number of *cis*-acting elements and activate the transposable elements. During allopolyploidization, the expression pattern of the *MBD* gene changes, which may be regulated by *cis*-acting elements and transposable elements. The number imbalance of *cis*-acting elements and transposable elements in A_n_ and C_n_ subgenomes may also lead to biased A_n_ subgenome expression of the *MBD* gene in *B. napus*.

**Conclusions:**

In this study, by evaluating the number, structure, phylogeny and expression of the *MBD* gene in *B. napus* and its diploid ancestors, we increased the understanding of *MBD* genes in allopolyploids and provided a reference for future analysis of allopolyploidization.

**Supplementary Information:**

The online version contains supplementary material available at 10.1186/s12870-022-03485-0.

## Background

Frequent and recurrent polyploidization events promote speciation and exist widely in the evolutionary history of plants [1–4]. Polyploidy provides the potential for genetic diversity while increasing the cost of plant genome replication and epigenetic instability [5]. Genomic changes caused by polyploidization are mostly nonadditive, such as genome reorganization or functional modifications [6]. Although gene expansion occurs from time to time during the process of polyploidization [7], the polyploid genome is not simply a direct doubling of diploid genomes. Extreme changes in the genome during the early stage of the formation of neopolyploids may lead to events such as DNA sequences and gene loss [8]. Gene expansion caused by polyploidization may eventually return to one gene copy through separation and loss, or it is possible that multiple copies of one gene develop different functional trends [2]. In addition, allopolyploid plants rely on the richness of their genomes, which has a significant impact on trait diversification, crop domestication, and environmental adaptation [4, 9, 10].

As an epigenetic modification, DNA methylation plays a significant role in gene expression regulation and genome stability [11]. The most well-known modifications of DNA-recognition domains are the SET and RING finger-associated domain (SRA) and the Methyl-CpG-Binding Domain (MBD) [12]. MBD proteins usually recognize symmetrically CG-methylated oligonucleotides, but studies have shown that they can also bind to mCA sites [13]. The importance of MBD proteins has led to heated research on their structure and function. It was found that MBD5 and MBD6 in *Arabidopsis thaliana* can be recruited into chromatin through the recognition of CG methylation sites, repression of gene subsets and transposons, and the maintenance of unchanged DNA methylation levels [13]. MBD7 protects genes from expression repression and DNA hypermethylation by binding to the highly methylated CG region and recruiting histone acetyltransferases [14]. MBD9 strongly interacts with Imitation SWItch (ISWI) chromatin remodeling complexes and has been shown to be involved in DNA demethylation [15–17]. In Arabidopsis mutants with *MBD* gene knockdown, nucleolar dominance was disturbed, and their growth and development were abnormal [18]. In mammals, MBD proteins are involved in tumorigenesis, schizophrenia, pulmonary fibrosis and many other diseases [19, 20].

The *Brassica* genus has excellent crop characteristics and a profound cultivation history. They experienced an additional whole-genome triplication (WGT) event after differentiation from Arabidopsis [21, 22]. Following WGT, gene loss events resulted in three subgenomes of the least fractionated blocks (LF), the medium fractionated blocks (MF1) and the most fractionated blocks (MF2) in *B. rapa* [23]. *Brassicaceae* share the same ancestral karyotypes, basic genomic blocks, and significant synteny between genomes [24]. *Brassica napus* (AACC, 2n = 4x = 38), an allotetraploid plant, is formed by interspecific crosses between two diploid ancestors, *Brassica rapa* (AA, 2n = 2x = 20) and *Brassica oleracea* (CC, 2n = 2x = 18), followed by whole-genome duplication (WGD) events and it is an excellent material for studying plant polyploidy [25]. To date, *MBD* genes have been identified and studied in Arabidopsis, rice, maize and other plants [26], but the influence of the process of allopolyploidy on this gene family is still unknown in the *Brassica* genus. In this study, based on the Arabidopsis MBD protein sequence, MBD proteins and genes were identified in *B. rapa*, *B. oleracea*, and *B. napus*. We explored the evolutionary experience of the *MBD* gene family and enhanced the understanding of the impact of allopolyploidization by analyzing the chromosome location, phylogenetic tree, syntenic genes, gene structure, evolutionary pressure, *cis*-acting elements, and gene expression patterns of *MBD* genes.

## Results

### Identification of the *MBD* genes

To accurately identify *MBD* gene family members, we considered that only those with complete MBD domains were MBD proteins. Thirteen putative MBD proteins in *A. thaliana* [26] were used as query sequences, and BLASTp was used to search for *B. rapa*, *B. oleracea*, and *B. napus* in the BRAD database (http://brassicadb.cn/#/). All of the identified candidate protein sequences were confirmed by the CDD, Pfam and InterPro databases, and the proteins with complete MBD domains that could be queried in all three databases were retained. Finally, ten genes encoding the MBD proteins were identified in *B. rapa*, eleven in *B. oleracea* and twenty-two in *B. napus*. Based on their homology with Arabidopsis *MBD* genes, they were named *BrMBD2* to *BrMBD11b* in *B. rapa*, *BoMBD2* to *BoMBD11* in *B. oleracea*, and *BnCMBD2a* to *BnAMBD11d* in *B. napus*. When homologous to the same Arabidopsis gene, the last letter of the name, from “a” to “g”, indicates a gradual decline in homology, and in *B. napus*, the A and C letters after “Bn” indicate the *B. napus* A_n_ subgenome and C_n_ subgenome. The total number of *MBD* gene family members in diploid ancestors was less than that in tetraploid *B. napus*, indicating expansion events of the *MBD* gene during the formation and evolution of *B. napus*.

### Chromosomal location and duplication pattern analysis of *MBD* genes

**Fig. 1:**
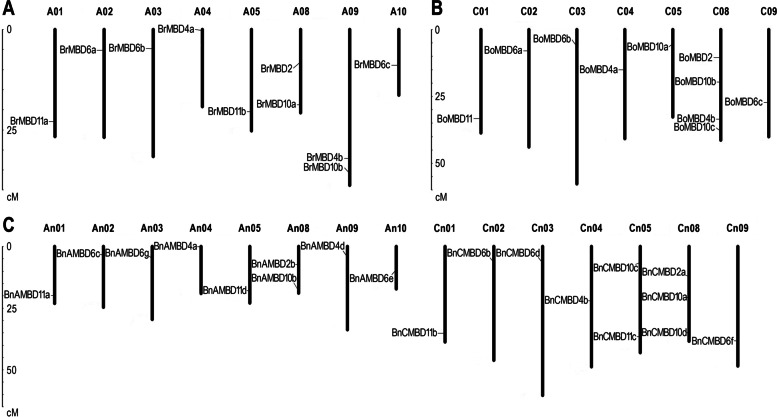
Chromosome distribution of *MBD* genes in *B. napus* and its diploid ancestors. Draw a Chromosome distribution based on the relative position of the gene on the chromosome in *B. rapa* (**A**), *B. oleracea* (**B**) and *B. napus* (**C**). *BnCMBD4c*, *BnCMBD6a* and *BnAMBD10e* of *MBD* genes in *B. napus* and *BoMBD9* in *B. oleracea* were located on the unassembled scaffolds. So they were not shown in the figure. Chromosome names were at the top and length scales were at the left

The locations of the *MBD* genes of *B. napus* and its diploid ancestors on the chromosomes were displayed using MapInspect software. In addition to *BnCMBD4c*, *BnCMBD6a* and *BnAMBD10e* in *B. napus* and *BoMBD9* in *B. oleracea*, which were located on the unassembled scaffold, the remaining 39 *MBD* genes were located on the chromosomes of each species, respectively (Fig. 1). Ten *B. rapa MBD* genes were located on 8 chromosomes (Fig. 1A), ten *B. oleracea MBD* genes were located on 7 chromosomes (Fig. 1B), and nineteen *B. napus MBD* genes were located on 15 chromosomes (Fig. 1C). Comparing the distribution of *MBD* genes on the *B. napus* A_n_ subgenome with the *B. rapa* A genome, the gene homology and their relative position on the chromosome were the same, except that A_n_09 may have gene loss or incomplete chromosome assembly. Similarly, comparing the gene distribution of the *B. napus* C_n_ subgenome with the *B. oleracea* C genome, one gene was added to C_n_05, a gene on C_n_08 was not located, and the remaining genes did not change their positions on the chromosome during the formation of *B. napus*. Relatively speaking, the A_n_ subgenome was more stable than the C_n_ subgenome, but overall, the *MBD* gene distribution on the chromosome did not change significantly during the process of hybridization and polyploidization.

More than one WGD event occurred during the formation of the ancestral polyploidy *Brassica* genus, and the formation of *B. rapa* and *B. oleracea* genomes was inseparable from whole genome triplication and extensive diploidization [27]. During evolution, the maintenance of gene family members often depends on different duplication modes [28]. We counted the members of the *MBD* genes according to the five duplication modes, i.e., WGD, TD (tandem duplication), PD (proximal duplication), TRD (transposed duplication), and DSD (dispersed duplication) and found that WGD, TRD and DSD, especially WGD and TRD, played a major role in the increase in *MBD* gene numbers in *B. rapa*, *B. oleracea* and *B. napus* (Additional file 1: Table S1). WGD events played an important role in *B. rapa* (8/10) and *B. oleracea* (9/11), but the importance of its tetraploid offspring in *B. napus* was reduced (11/22). In contrast, DSD events were slightly more frequent in *B. napus* (20/22) than in *B. rapa* (8/10) and *B. oleracea* (9/11). The proportion of TRD events in *MBD* gene duplication in *B. napus* was similar to that of its two ancestor species. TD and PD were not detected in the three species, suggesting that *MBD* genes in *B. rapa*, *B. oleracea* and *B. napus* may not be affected by these two events. A study showed that genes increased by WGD were often accompanied by TRD and DSD events [29], which was consistent with what we found in the *MBD* gene family. TRD may be a long-distance gene transposition through DNA-based or RNA-based transposable elements (TEs) [30]. Therefore, we predicted the TEs within 2000 bp upstream and downstream of the *MBD* genes (Additional file 2: Table S2). We found 83, 88 and 182 TE fragments in *B. rapa*, *B. oleracea* and *B. napus*, respectively. These results showed that the number of TEs of *MBD* genes, especially DNA transposons, was significantly increased compared with diploid ancestors in *B. napus*. Gene duplication rapidly provides a large number of options for natural selection.

### Phylogenetic analysis of *MBD* genes

**Fig. 2:**
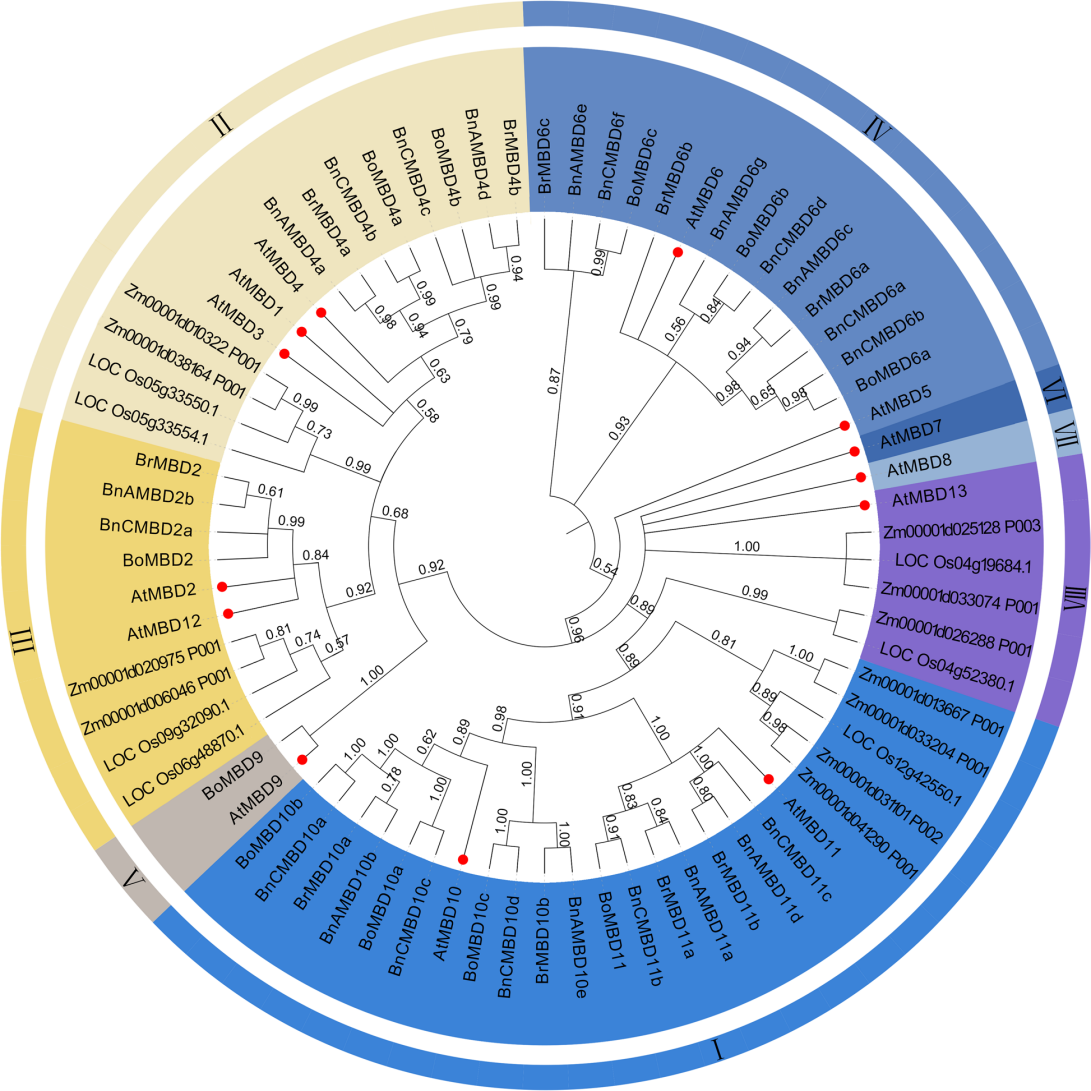
Phylogenetic tree of *MBD* gene family in 6 species. *MBD* genes from four dicotyledons (*B. rapa*, *B. oleracea*, *B. napus* and *A. thaliana*) and two monocotyledons (*Oryza sativa* and *Zea mays*) were displayed in the phylogenetic tree. In the MEGA X software, using the Maximum Likelihood (ML) and 1000 bootstrap repeats, seventy-four protein sequences were analyzed to construct a phylogenetic tree. The phylogenetic tree was divided into 8 clades, and different colors represented different clades. The figure only showed bootstrap values greater than 50%, and 13 *A. thaliana MBD* genes were marked with red dots

To elucidate the phylogenetic and evolutionary relationships among *MBD* genes, we constructed a phylogenetic tree based on 74 protein sequences from four dicotyledons (*B. rapa*, *B. oleracea*, *B. napus* and *A. thaliana*) and two monocotyledons (*Oryza sativa* and *Zea mays*, Fig. 2). The MBD protein sequences of Arabidopsis were obtained from the TAIR database, and the protein sequences of rice, maize, *B. rapa*, *B. oleracea* and *B. napus* were identified according to the same criteria. Taking typical monocotyledons and dicotyledons representing Arabidopsis, rice and maize whose *MBD* gene family had been studied in detail as an outgroup, a phylogenetic tree was constructed together with *B. rapa*, *B. oleracea* and *B. napus* to make the result more accurate and show the evolutionary direction. Based on the clades of the *MBD* gene in Arabidopsis, rice and maize, we also divided the phylogenetic tree containing *B. rapa*, *B. oleracea* and *B. napus* into 8 clades, including 24 genes of clade I, 15 genes of clade II, 10 genes of clade III, 15 genes of clade IV, 2 genes of clade V, 1 gene of clade VI, 1 gene of clade VII and 6 genes of clade VIII. Among them, the true *MBD* genes in *Brassica* only existed in clades I to V. In this study, *MBD* genes of monocotyledons, maize and rice, were located in clades I, II, III and VIII, while dicotyledons were distributed in all eight clades. What attracted special attention was that monocotyledons have been confirmed to have no *MBD* genes in clade IV and clade VI [26]. Monocotyledons and dicotyledons seem to have appeared in the early Cretaceous as the main clades of angiosperms [31]. We found that the *MBD* genes had a long evolutionary history. As early as the formation period of monocotyledons and dicotyledons, the *MBD* genes already existed and began to differentiate. In addition, the *MBD* genes maintained good homology during long evolution among *B. rapa*, *B. oleracea*, *B. napus*, *Oryza sativa* and *Zea mays*. *MBD* genes with homology to the same *AtMBD* were located in the same clades. However, at the same time, some *MBD* genes may have lost homologous genes or changed their structure during evolution; for example, *AtMBD7* and *AtMBD8* lack closely related branches on the phylogenetic tree. Considering their structural integrity, some *MBD* genes in *Oryza sativa*, *Zea mays*, *B. rapa*, *B. oleracea* and *B. napus* may not be discussed.

### Analysis of *MBD* genes structure and GO enrichment

**Fig. 3:**
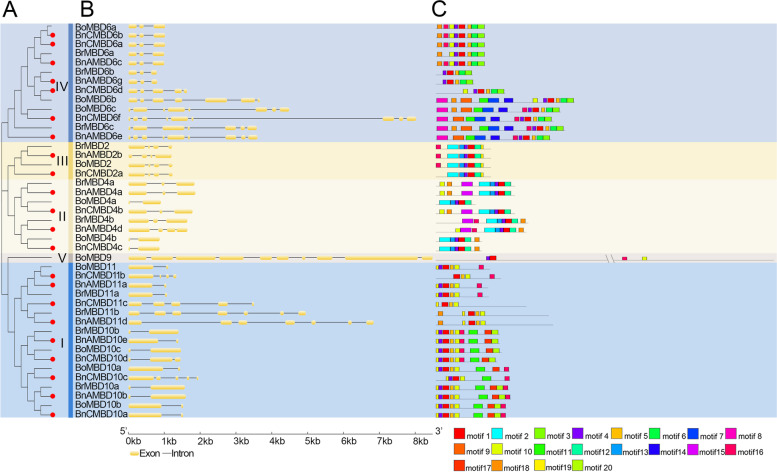
Analysis of intron/exon and conserved motif characterizations of *MBD* genes. **A** Phylogenetic trees of *B. rapa*, *B. oleracea* and *B. napus*. **B** Introns and exons of the *MBD* genes. **C** Conserved motifs of MBD proteins. Different background colors corresponded to the colors of different clades, whose names were on the right side of the phylogenetic tree (**A**). The *MBD* genes in *B. napus* on the left phylogenetic tree were marked with red dots

We constructed another phylogenetic tree for the *MBD* genes of *B. rapa*, *B. oleracea* and *B. napus* and analyzed exon-intron structures to explore the effect of allopolyploidization on the *MBD* gene structure (Fig. 3). The topological structure of the two phylogenetic trees was consistent, but the analyzed species were not completely the same. The latter tree only contained members of the *Brassica MBD* gene family. *B. napus* and its diploid ancestors did not have corresponding *MBD* gene family members in clades VI, VII and VIII, so only clades I to V are shown (Fig. 3A). The same clade used the same background color in both figures and contained the same *MBD* genes of *B. rapa*, *B. oleracea* and *B. napus*. The results showed that the number of introns varied significantly among *MBD* genes, ranging from 1 to 9 (Fig. 3B). Among them, the number of introns in clade II and clade III is relatively small, containing only 1 to 3 introns. We believe that two genes from the terminal branch of the same phylogenetic tree, if one came from *B. napus* and the other from either *B. rapa* or *B. oleracea*, were considered to have a direct evolutionary relationship. Seventeen out of twenty-two *B. napus* genes were found to be directly related to their parents. Of the 17 gene pairs, 10 had the same number of introns, and 7 had an increase. The phenomenon of increasing intron numbers was widespread.

To further understand the potential function of *MBD* genes in *B. rapa*, *B. oleracea* and *B. napus*, we conducted Gene Ontology (GO) enrichment analysis for the three species (Additional file 3: Fig. S1). In *B. rapa* (Additional file 3: Fig. S1A) and *B. oleracea* (Additional file 3: Fig. S1B), *MBD* genes were mainly enriched in molecular functions, especially in these three terms: “methyl-CpG binding”, “nucleotide binding” and “nucleoside phosphate binding”. In *B. napus* (Additional file 3: Fig. S1C), in addition to being abundantly enriched in the above three molecular functions as in their diploid ancestors, various cellular components represented by “perinucleolar chromocenter” and “nucleolus organizer region” were also enriched.

### Prediction of the amino acid sequence and physicochemical properties of the MBD protein

We also analyzed the conserved motifs of 43 proteins encoded by *MBD* genes (Fig. 3C). Similarly, most of the clades had similar motif types. However, overall, there was a large gap between the number and type of conserved motifs in the different MBD proteins. Motif 1 and motif 4 existed in almost all MBD proteins. Notably, motif 3 and motif 6 in clade IV were present in every MBD protein and only in this clade. These two motifs may have special functions, distinguishing the proteins of clade IV from the MBD proteins of other clades.

**Fig. 4:**
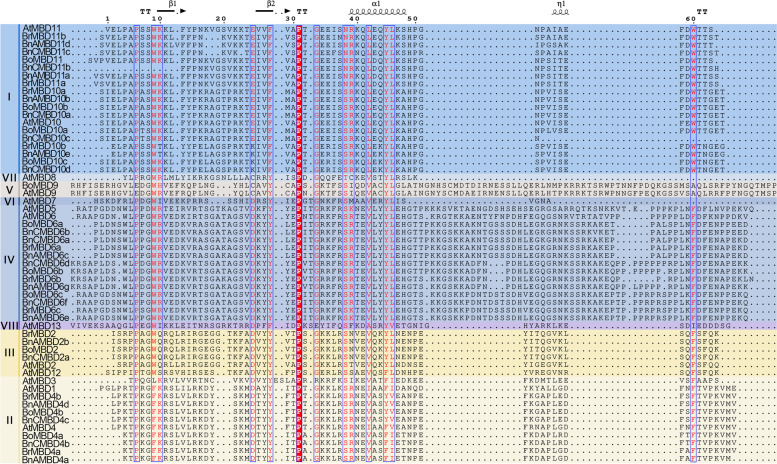
Sequence alignment of MBD proteins conserved domains. The conserved domain sequences of MBD proteins in *B. rapa*, *B. oleracea*, *B. napus* and *A. thaliana* were compared with ClustalW. Different background colors represented different clades whose names were on the left side. Secondary structures were shown at the top of the figure, with α and β meaning helix and fold respectively, η meaning 3_10_-helix, arrow meaning β-strand, and TT meaning strict β-turn. The blue box enclosed the sites with high similarity

To further understand the structural characteristics of MBD proteins, we compared the domain sequences of *B. rapa*, *B. oleracea*, *B. napus* and *A. thaliana* (Fig. 4). The blue line framed the sites with high similarity. The top of the figure shows the secondary structure of the protein, and the right side shows the name of the different clades of the phylogenetic tree (Fig. 2). In Figs. 2, 3 and 4, the same background colors represented the same clades. The corresponding clades of *Brassica* and *A. thaliana MBD* gene family members in Figs. 2 and 4 remained unchanged. There were multiple MBD domains in AtMBD7, and only the domain sequence with a complete structure is shown in the figure. The MBD domains contained α helix and β fold. However, the MBD domain is a hallmark feature of the MBD protein. These results showed that the overall conservation of MBD protein domains was not high, and there were great differences among different clades, but some sites were highly conserved, which may be related to different MBD proteins performing different functions.

We used the ProtPram tool on the ExPASy server to predict the physical and chemical properties of 43 MBD proteins, such as molecular weight (MW), isoelectric point (pI), grand average of hydropathicity (GRAVY) and instability index online (Additional file 4: Table S3). The results showed that among the MBD proteins in *B. napus*, the average protein length (332.4 aa) was lower than that of its diploid ancestors *B. rapa* (348.3 aa) and *B. oleracea* (500.7 aa). Correspondingly, the average molecular weight of MBD proteins in *B. napus* was 36.71 kDa, which was lower than that of *B. rapa* (38.62 kDa) and *B. oleracea* (55.31 kDa). The pI of *B. rapa*, *B. oleracea* and *B. napus* varied from 4.60 to 9.56, and more than half of them were less than 7. Except for BoMBD10a, the remaining MBD proteins were all classified as unstable (instability index > 40). Forty-three MBD proteins were all hydrophilic proteins. Using WoLF PSORT software to predict the subcellular location of MBD proteins, the results showed that most MBD proteins in *B. napus* were located in the same location as their ancestral homologs in the nucleus.

### Synteny analysis of *MBD* genes

**Fig. 5:**
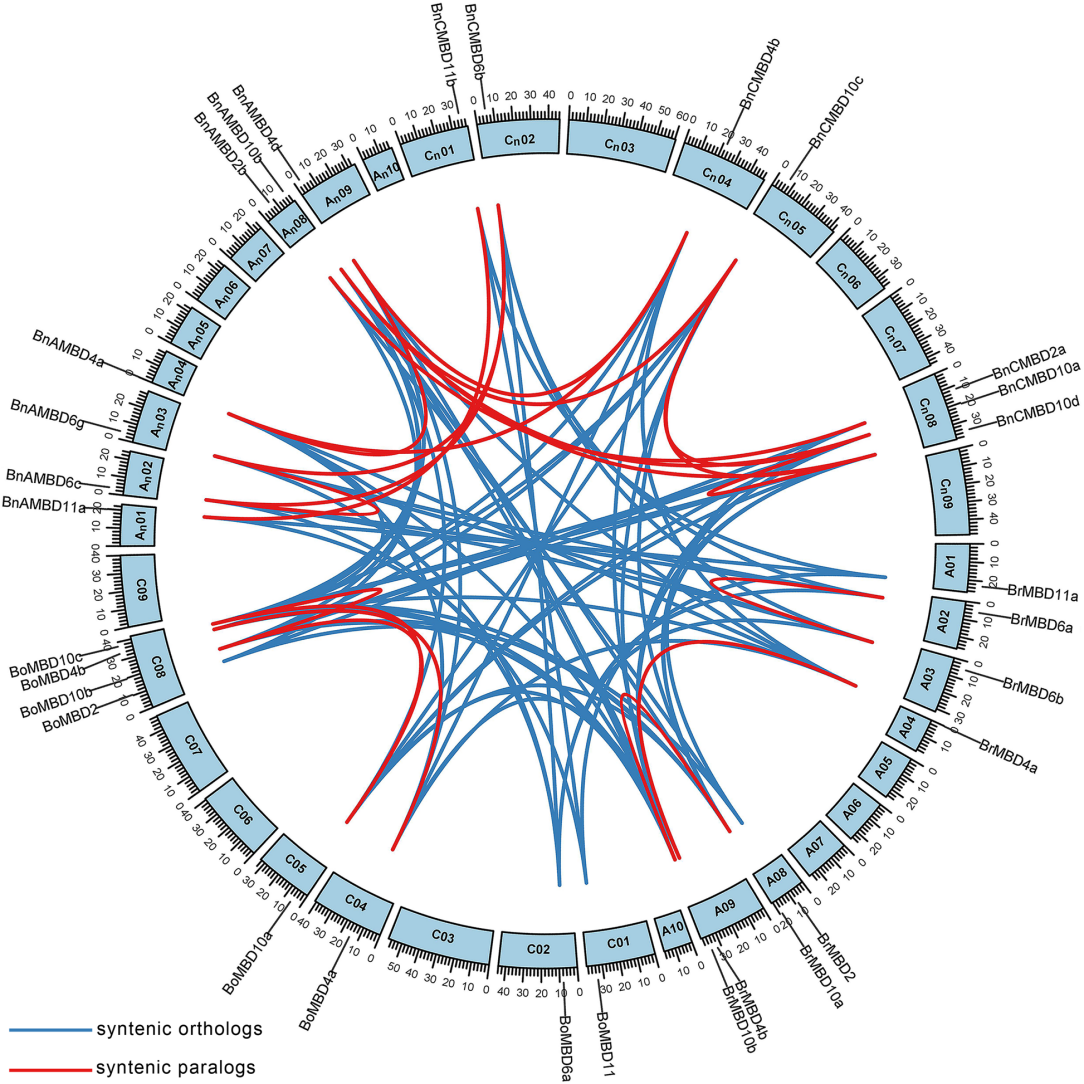
Genome-wide synteny analysis for *MBD* genes in *B. rapa*, *B. oleracea* and *B. napus*. *B. rapa* chromosomes were A01-A10, *B. oleracea* chromosomes were C01-C09, *B. napus* A_n_ subgenomic chromosomes were A_n_01-A_n_10, *B. napus* C_n_ subgenomic chromosomes were C_n_01-C_n_09. All the syntenic genes identified in the *MBD* genes were mapped to the responding chromosomes, except for the 9 gene pairs that had syntenic relationships with the *BnAMBD10e* gene located to the unassembled scaffold, which were not shown in the figure. Blue lines linking the syntenic orthologs, and red lines linking the syntenic paralogs

We used the BRAD database to query the syntenic relationship among the *MBD* genes and to draw a synteny map between *B. napus* and its diploid ancestors (Fig. 5, Additional file 5: Table S4). Two syntenic genes are connected by an arc, with the blue lines linking the between species, and the red lines linking the syntenic paralogs (Fig. 5). A total of 68 pairs of syntenic orthologs and 25 pairs of syntenic paralogs were identified in *B. napus* and its diploid ancestors. Among them, *BnAMBD10e* was located on the unassembled scaffold, so nine syntenic gene pairs associated with it are not shown in the figure. *MBD* genes of *B. napus* had strong synteny with those of its ancestors. Approximately 72.1% of *MBD* gene family members were located in the syntenic region, and syntenic genes were widely distributed in the genomes of the three species. The syntenic genes corresponding to *AtMBD2* and *AtMBD12* were the same in *B. rapa*, *B. oleracea* and *B. napus*. Fourteen syntenic gene pairs were found between *B. rapa* and *B. oleracea*, while only twelve syntenic gene pairs were found between the A_n_ and C_n_ subgenomes of *B. napus*, suggesting that *MBD* syntenic genes loss may occur during allopolyploidization. It is worth mentioning that only *AtMBD2*, *AtMBD4*, *AtMBD6*, *AtMBD10*, *AtMBD11* and *AtMBD12* corresponding syntenic genes were found in *B. rapa*, *B. oleracea* and *B. napus*, while *AtMBD1*, *AtMBD3*, *AtMBD5*, *AtMBD7*, *AtMBD8*, *AtMBD9* and *AtMBD13* were not found. This is probably caused by the loss of some copies of these genes or the changes in their gene structures during the process of evolution, which were not identified as real *MBD* genes in this study.

We calculated the nonsynonymous (Ka) and synonymous (Ks) substitution values and Ka/Ks ratios of duplicated gene pairs in *B. napus* and its ancestors to characterize the selection pressure on *MBD* genes during evolution (Table 1). Generally, the Ka/Ks ratio is used to represent the selection pressure on genes. A ratio greater than 1 means positive selection, less than 1 means purifying selection, and equal to 1 means neutral selection [32]. The results showed that there were no duplicated gene pairs between *B. rapa* and *B. oleracea*. The Ka/Ks ratios of the eight pairs of duplicated genes in the subgenomes of *B. napus* were less than 1, which indicated that they underwent purifying selection.

**Table 1:** Ka/Ks ratios and selection types of *MBD* duplicated gene pairs

Duplicated gene pairs	Ka	Ks	Ka/Ks	Types of selection
*BnCMBD2a*-*BnAMBD2b*	0.009	0.059	0.146	Purify selection
*BnAMBD4a*-*BnCMBD4b*	0.015	0.058	0.250	Purify selection
*BnCMBD6a*-*BnCMBD6b*	0.006	0.037	0.158	Purify selection
*BnCMBD6a*-*BnAMBD6c*	0.012	0.037	0.319	Purify selection
*BnCMBD6b*-*BnAMBD6c*	0.018	0.075	0.234	Purify selection
*BnAMBD6e*-*BnCMBD6f*	0.010	0.065	0.155	Purify selection
*BnCMBD10a*-*BnAMBD10b*	0.039	0.143	0.270	Purify selection
*BnCMBD10d*-*BnAMBD10e*	0.042	0.067	0.626	Purify selection

### Analysis of *cis*-acting elements in the *MBD* genes

**Fig. 6:**
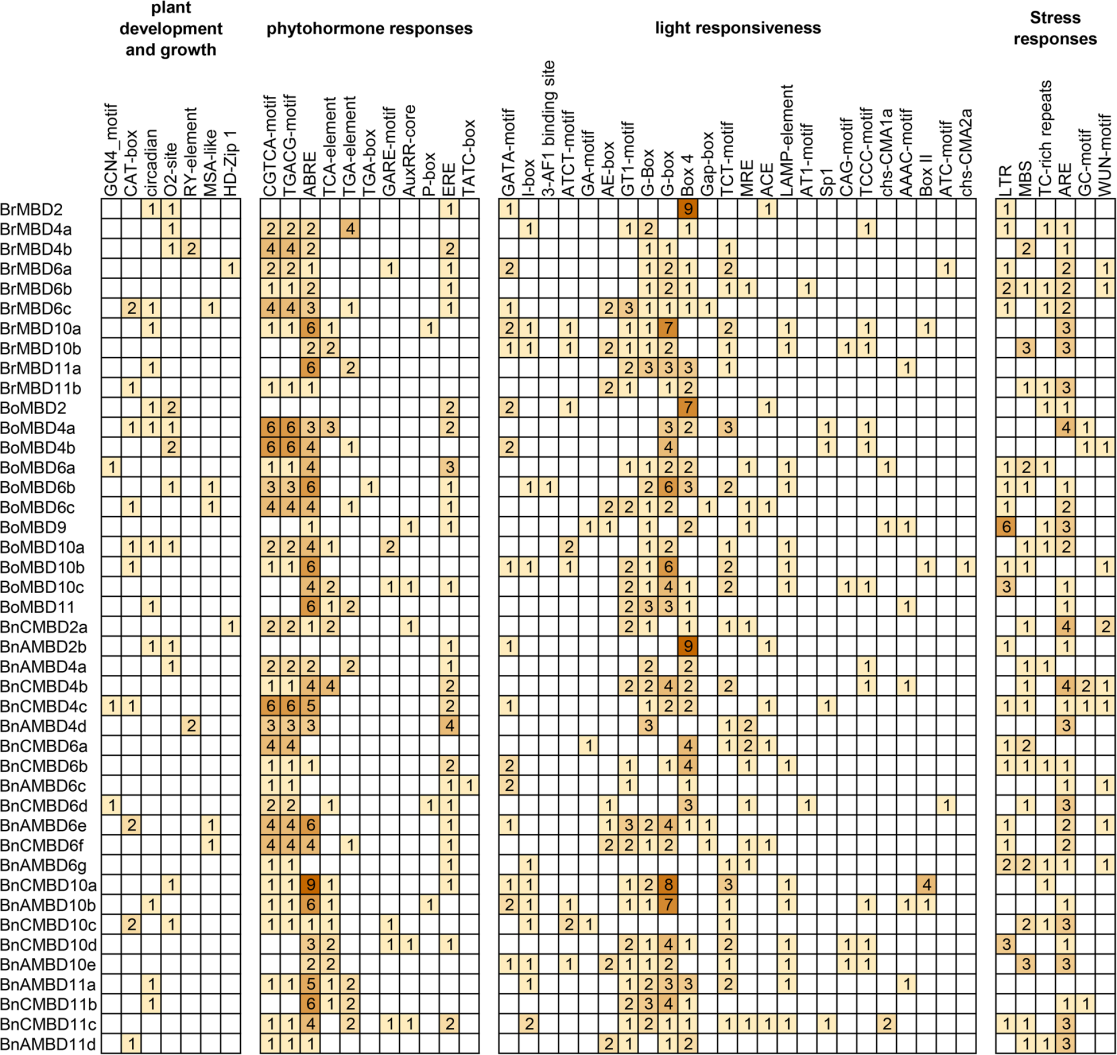
*Cis*-acting elements in the promoter regions of the *MBD* genes

Different *cis*-acting elements in gene promoters may be related to different functions of genes. To identify *cis*-acting elements of *MBD* genes, gene sequences within 1500 bp upstream of the gene transcription start site (TSS) were extracted and retrieved using PlantCARE [33]. The results showed that the upstream promoter regions of the *MBD* genes contained a large number of *cis*-acting elements in *B. napus* and its diploid ancestors. Figure 6 shows the *cis*-acting elements associated with four types of life activities: plant development and growth (7), phytohormone responses (11), light responsiveness (24) and stress responses (6). Among them, there were abundant *cis*-acting elements related to phytohormone responses and light responsiveness, especially the CGTCA-motif and TGACG-motif involved in MeJA-responsiveness, ABRE involved in abscisic acid responsiveness, and the GT1-motif, G-Box, G-box and Box 4, which are related to light responsiveness. A large number of them were found in the identified *MBD* promoter regions. In addition, ARE related to anaerobic induction made up an important proportion of the stress response elements. The three species all contained a large amount of ARE, and the number in *B. napus* was significantly greater than the sum of its ancestor species. Overall, the number of *cis*-acting elements found in *B. napus* (517) was roughly the same as the sum found in *B. rapa* (219) and *B. oleracea* (294). During the process of allopolyploidization, the number of *cis*-acting elements related to plant development and growth decreased, but the other three types increased, especially *cis*-acting elements related to stress, which may be related to the stronger resilience of tetraploid plants.

### Gene expression pattern analysis of *MBD* genes

**Fig. 7:**
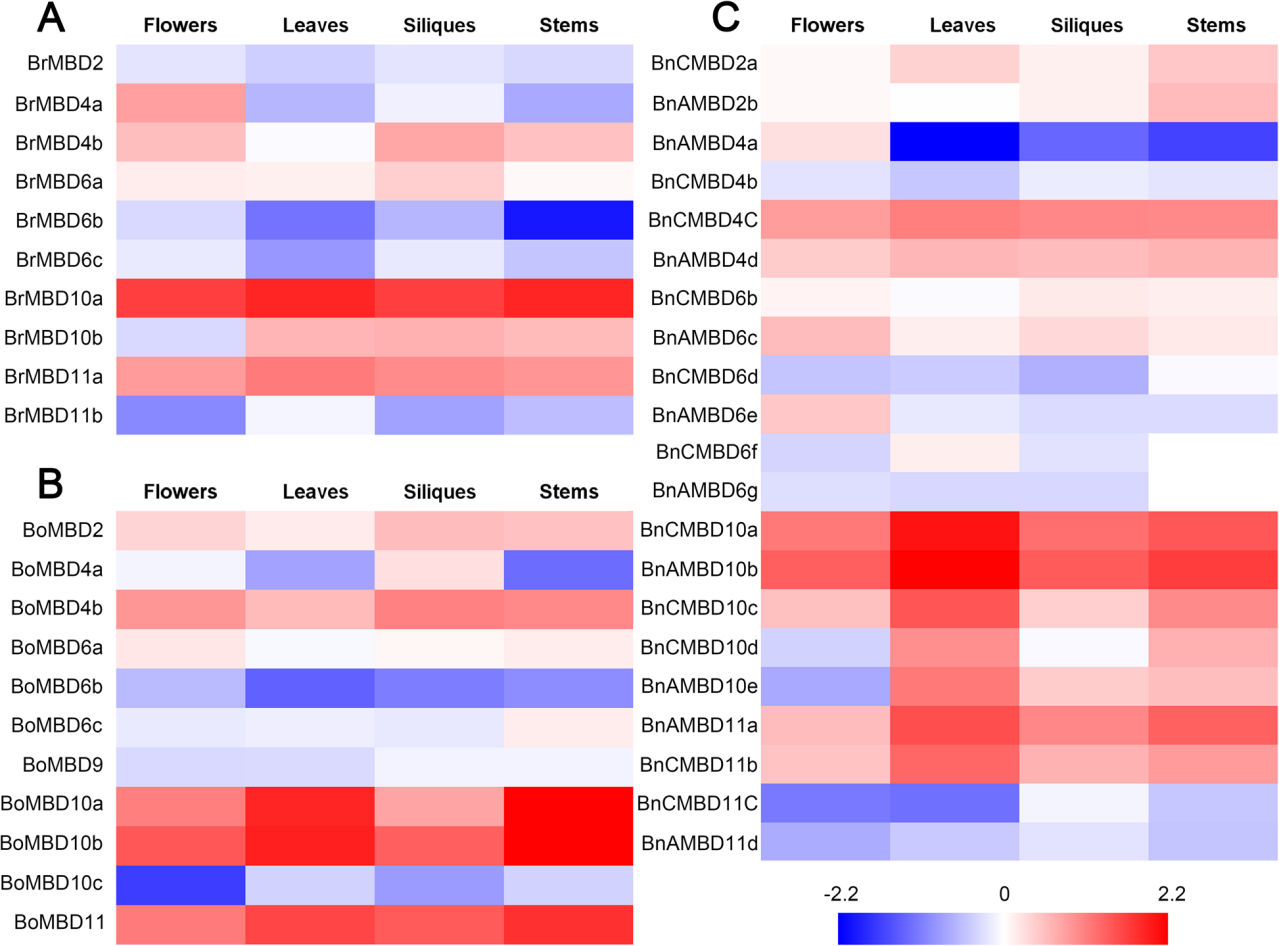
The expression of *MBD* genes in the flowers, leaves, siligues and stems. Expression patterns of *MBD* genes in *B. rapa* (**A**), *B. oleracea* (**B**) and *B. napus* (**C**). Red represented high expression and blue represented low expression

We used flowers, leaves, siliques and stems to analyze the expression patterns of *MBD* genes in the three species (Fig. 7, Additional file 6: Table S5). Overall, although there was a significant difference in expression among the genes, the specificity between tissues was weak. However, the expression of different genes between tissues still had a certain preference. For example, the expression level of *BnCMBD10c* in leaves was significantly higher than that of the other three tissues, but the expression of *BnCMBD11c* was the opposite. The number of clade I genes (Fig. 2) of all three species (except *BrMBD11b*, *BoMBD10c*, *BnCMBD11c* and *BnAMBD11d*) showed high expression levels. In contrast, the gene expression levels of clade IV were relatively low in all species, and *BnCMBD6a* was not expressed in any of the four tissues. The difference in expression levels between different clades may be a manifestation of the diverse functions of *MBD* genes.

To explore whether the expression patterns of *MBD* genes changed among the four tissues during allopolyploidization, we compared the expression patterns between gene pairs with direct evolutionary relationships (Fig. 3A). The expression patterns of most gene pairs changed. For example, *BrMBD2* was expressed at a low level in the four tissues, while *BnAMBD2b* was expressed at a high level in the stems. *BoMBD4a* was highly expressed in siliques and low in leaves and stems, but the expression level of *BnCMBD4b* was almost the same among the four tissues. *BoMBD10c* had low expression levels in the four tissues, especially in flowers. *BnCMBD10d* expression was low in flowers, but it had high expression levels in leaves and stems. Interestingly, only one of the eight pairs of duplicated genes (Table 1) shared the same expression pattern. This may further indicate that functional differentiation of the *MBD* genes occurred during evolution.

We used FPKM (fragments per kilobase million) values to analyze the expression bias of 17 gene pairs with direct evolutionary relationships (Additional file 7: Table S6). Because the homologous genes of *MBD2* existed in only one parent, it was not discussed. Different genes had different expression biases in tissues. The expression of *MBD4* in leaves was biased toward *B. rapa* and biased toward *B. oleracea* in the other three tissues. *MBD6* expression in leaves and siliques was biased toward *B. oleracea*, and it was biased toward *B. rapa* in flowers and stems. The expression of *MBD10* was biased toward *B. oleracea* in flowers and siliques and toward *B. rapa* in leaves and stems. The expression of *MBD11* was biased to *B. rapa* in all four tissues. Overall, for *MBD* genes with expression bias, most genes were biased toward *B. oleracea* in siliques and *B. rapa* in leaves and stems, while the number of genes biased toward *B. rapa* and *B. oleracea* in flowers was the same.

## Discussion

With the significant increase in the number of genome-sequenced plants, the understanding of polyploidy evolution has moved from a “dead end” to today’s “contemporary innovation” [34]. Polyploidy is common in angiosperms, and WGD events contribute to the dominance of angiosperms by providing important genes for seed and flower development [35]. Some gene families also change during this process, such as family expansion or contraction [36]. In *Brassica*, we also observed an expansion of this gene family, with more *MBD* genes identified in *B. napus* than in any other species previously, such as Arabidopsis, rice, maize, and wheat. The *Brassica* genus contains neotetraploidization and mesohexaploidization species and is an excellent model for studying polyploidy evolution [37]. The MBD protein participates in the DNA methylation process and is closely related to plant growth and development. The *MBD* gene family has been studied in Arabidopsis, rice, maize, and wheat [26, 38], but studies in *Brassica* have not yet been reported. We analyzed the *MBD* gene family in *B. rapa*, *B. oleracea*, and *B. napus* to understand the influence of allopolyploidization on this gene family.

### *MBD* gene sequence differentiation is not achieved overnight but gradually evolves

From the perspective of phylogenetic evolution, *MBD* genes of Arabidopsis, rice and maize can be divided into eight clades, each with its unique characteristics [26]. Arabidopsis *MBD* gene family members were derived from previous studies, so we did not delete even potential *MBD* genes. However, the five species reidentified in this study, *B. rapa*, *B. oleracea*, *B. napus*, *Oryza sativa* and *Zea mays*, were identified based on strict criteria. *MBD* genes were discussed only if the encoded MBD proteins contained a complete domain (Fig. 2). Therefore, the number of genes encoding the MBD protein of rice and maize was less than 17 and 14, respectively [39], in phylogenetic tree construction. In *B. rapa*, *B. oleracea*, and *B. napus*, *MBD* gene family members were identified in only five clades of the phylogenetic tree, lacking three clades compared with Arabidopsis *MBD* gene family members in eight clades. This may be due to the loose identification criteria of Arabidopsis, used for constructing clades of potential *MBD* genes. After *Brassica* and Arabidopsis separated, some proteins encoded by the *MBD* genes in *B. rapa*, *B. oleracea*, and *B. napus* may be incomplete or lost domains during the process of independent evolution. Monocotyledons and dicotyledons are two aspects of the angiosperms. Clade IV and VI genes are unique to dicotyledons and are not found in rice and maize. This may indicate that clade IV and clade VI of *MBD* genes evolved independently in dicotyledon clades. Although most of the genes in each clade of the *MBD* gene family are related to DNA methylation and demethylation, only the proteins encoded by these two clades can still specifically bind to methylation sites in vitro [40]. The methyl-binding ability of MBD proteins is not achieved overnight but gradually in evolves. The remaining clade genes have much older evolutionary histories and may have more traditional functions during plant growth and development, although they have not been verified to have binding activity in vitro.

Syntenic gene pairs share the same ancestral genomic fragments, and they often have certain similarities in function, so the analysis of syntenic genes is very important for gene and genome research [24]. Syntenic and collinear gene pairs can be identified by searching for conserved blocks, allowing us to better understand the origin of genes. We found that *MBD* genes had great synteny. Syntenic gene pairs may indicate that *MBD* genes have different conservation levels during evolution. The syntenic genes corresponding to *AtMBD2*, *AtMBD4*, *AtMBD6*, *AtMBD10*, *AtMBD11* and *AtMBD12* may be more conserved and retain more features of their ancestral genes. Some genes with a weak relationship with synteny may be due to complete transposition and a loss of ancestors, or they may be ancient genes and not closely related to the others [28]. For the duplicated gene pairs with direct evolutionary relationships, we calculated the Ka/Ks values to evaluate the selection type (Table 1). After allopolyploidization, the Ka/Ks ratio between *MBD* genes in *B. napus* was less than 1, and the *MBD* genes were under strong purifying selection pressure. Purifying selection may restrict the spread of alleles or mutations so that most *MBD* genes can still retain the characteristics of their ancestor genes [41], which also verified their strong synteny. These genes still retained multiple pairs of duplicate genes in this case, which also proved that polyploidy may protect genes through rapid subfunctionalization [42]. This phenomenon has also been found in another gene family in *Brassica* [43].

In terms of gene structure, introns interrupt multiple exon combination sequences and generate mature mRNA through shearing [44]. In our study, the *MBD* genes were less conserved, and the phenomenon of intron acquisition generally occurred. Among the 17 pairs of genes with a direct evolutionary relationship, the number of introns in 10 pairs of genes remained unchanged, and 7 pairs of genes increased, while no intron reduction was found (Fig. 3B). Some researchers believe that introns provide advantages to plants, such as regulating gene expression and increasing protein diversity [45]. The phenomenon of intron increase has also appeared in other gene families in *B. napus* [46], indicating that the process of allopolyploidization may enhance the ability of plants to adapt to the environment through an increase in gene introns.

### Allopolyploidization increases the diversity of MBD protein motifs

The types of MBD protein motifs are abundant, but there are great differences between different clades (Fig. 3C). Motif 1 and motif 4 were highly conserved among MBD proteins and may be characteristic motifs of MBD proteins. Motif 3 and 6 were unique in clade IV and may be related to the specific function of clade IV. Similar phenomena were observed in the potato *MATE* gene family [47]. The GO enrichment results showed that *MBD* genes were mostly enriched in “methyl-CpG binding”, “nucleotide binding” and “nucleoside phosphate binding”, which was consistent with the known function of the *MBD* gene family. However, the difference in motifs among different clades, especially the existence of unique motifs in clades such as motif 3 and motif 6, may indicate functional divergence among *MBD* genes. Further analysis of the amino acid sequences showed that the MBD protein domains were less conserved, except for individual sites, but the protein within the same clade was highly conserved (Fig. 4). This may be caused by the process that endowed multicopy genes with different fates, in which many genes return to a single copy after extensive fractionation of polyploidy expanded homologous genes [2]. The conservation of the same clade among different species suggests that the *MBD* gene sequences may differentiate as early as the formation period of monocotyledons and dicotyledons, and further consolidate during polyploidy. In addition, the motifs of the proteins encoded by seven gene pairs with direct evolutionary relationships changed, although there was no one-to-one correspondence with gene pairs in which the intron increased. Compared with diploid ancestors, the numbers of these seven MBD protein motifs increased or decreased and the motif types changed in *B. napus*. This indicated that allopolyploidization also led to *MBD* gene sequencing and functional differentiation.

### *MBD* gene expression bias in allopolyploid *B. napus* may be regulated by TEs and *cis*-acting elements

Ancestral species usually provide two or more asymmetric subgenomes for allopolyploids [48]. The contribution of both parents to gene expression is not completely additive, and gene expression dominance has become a characteristic feature of many allopolyploid plants [49]. Homologous expression bias between subgenomes can promote the adaptability and flexibility of newly formed polyploids to the environment [50, 51]. In our study, the subgenomes of A_n_ and C_n_ retained the structure of ancestral chromosomes to some extent, and the position and number of the *MBD* genes on chromosomes is mostly unchanged (Fig. 1). However, the expression of some *MBD* genes is biased among multiple tissues (Additional file 7: Table S6), and more than half of the genes were biased in *B. rapa*, slightly more than *B. oleracea*.

The expression pattern of *MBD* genes changed significantly in *Brassica* during the allopolyploidization process. *Cis*-acting elements and TEs can regulate gene expression and change the gene expression patterns [52, 53]. We divided the *cis*-acting elements of *MBD* genes into four groups: plant development and growth, phytohormone responses, light responsiveness and stress responses. The number of *cis*-acting elements in *B. oleracea* was greater than that in *B. rapa*, and the number of *cis*-acting elements in *B. napus* was almost the same as the sum of the diploid ancestors. Interestingly, while the total amount remained the same, there were increases or decreases in different groups. *B. napus* may enhance its adaptability to the environment through the regulation of *cis*-acting elements. On the other hand, TE amplification may have driven the formation of the *B. napus* genome [54]. We identified 83, 88 and 182 TE fragments within 2000 bp upstream and downstream of the *MBD* gene in the *B. rapa*, *B. oleracea* and *B. napus* genomes, respectively. Compared with the diploid ancestors, the number of TE fragments in *B. napus* increased significantly, which may be activated by polyploidization [55]. Gene expression was affected by the number and distance of nearby TEs [56]. The subgenome with the greatest TE density had a relatively high probability of gene loss and inactivation and a weak overall expression level [57]. The number of TEs in *B. oleracea* was slightly greater than that in *B. rapa*, which may also provide an explanation for the bias of *MBD* gene expression in *B. napus*.

## Conclusions

In this study, 10, 11 and 22 *MBD* genes were identified in *B. rapa*, *B. oleracea* and *B. napus*, respectively. The WGD, TRD and DSD events maintained the number of *MBD* gene family members in these three species, and there was a strong syntenic relationship among these genes. The Ka/Ks ratios of duplicated genes were less than 1, and intense purification pressure may promote *MBD* genes to retain genes through sequence and functional differentiation. Sequence differentiation of *MBD* genes has been occurring continuously since the formation period of monocotyledons and dicotyledons, and each of the eight clades has its own characteristics. During the process of allopolyploidization, the *MBD* genes retained their original structural features and also showed some new changes, such as an increase in intron number in *B. napus*. Meanwhile, MBD proteins in different clades had their own motifs, and the number and type of motifs changed during the allopolyploidization process. This process may increase the number of *cis*-acting elements and activate TEs. In *B. napus*, *cis*-acting elements and TEs may regulate the expression of *MBD* genes to improve the plant’s ability to adapt to the environment. At the same time, their number imbalance in the A_n_ and C_n_ subgenomes may lead to *MBD* gene expression bias in *B. napus*. These results can enhance the understanding of the *MBD* gene family and provide a reference for future exploration of allopolyploids.

## Materials and methods

### Identification of *MBD* genes

In this study, thirteen potential *A. thaliana* MBD protein sequences were obtained from TAIR database (https://www.arabidopsis.org/) and used as query sequences for BLASTp searches (E-value < 10^− 5^) of proteins in *B. rapa*, *B. oleracea* and *B. napus* in the BRAD database (http://brassicadb.cn/#/) [58]. To identify the true MBD proteins, only the proteins containing the complete MBD domain were identified as MBD proteins. After the repetitive values were removed, all the identified candidate protein sequences were confirmed by CDD (https://www.ncbi.nlm.nih.gov/Structure/cdd/wrpsb.cgi) [59], Pfam (http://pfam.xfam.org/) [60] and InterPro (https://www.ebi.ac.uk/interpro/search/sequence/) [61] databases, and the proteins with complete MBD domains that could be queried in all three databases were retained. Finally, the identified *MBD* genes were named based on their homology with *MBD* genes in *A. thaliana*. Using the same standards and methods, the MBD proteins in *Oryza sativa* and *Zea mays* were identified in the public database Phytozome (https://phytozome-next.jgi.doe.gov/), and the required genomic data were obtained [62].

### Characterization of the MBD proteins

Physical and chemical properties of MBD proteins such as molecular weight (MW), grand average of hydropathicity (GRAVY), instability index and isoelectric point (pI) were predicted online using ProtPram tool on ExPASy server (https://www.expasy.org/) [63].The WoLF PSORT (https://wolfpsort.hgc.jp/) [64] online tool predicts subcellular localization of MBD proteins.

### Chromosome location and gene structure analysis

The location information of *MBD* genes of *B. rapa*, *B. oleracea* and *B. napus* were obtained from BRAD database, and the gene chromosome location diagram was drawn by MapInspect software. The coding sequences (CDS) were analyzed on the Gene Structure Display Server (GSDS) 2.0 (http://gsds.gao-lab.org/) [65], and the exon/intron structures of the *MBD* genes were displayed.

### Identification of TEs and *cis*-acting elements

The 1500 bp upstream sequences of *MBD* genes were extracted from BRAD database, and Plant *Cis*-Acting Regulatory Element (PlantCARE) website (http://bioinformatics.psb.ugent.be/webtools/plantcare/html/) was used to search and analysis *cis*-acting elements in promoter regions and classified them according to their functions [33]. TEs within 2000 bp upstream and downstream of *MBD* genes were predicted in database Repbase Update (RU, https://www.girinst.org/) [66].

### Gene duplication and synteny analysis

The online database Plant Duplicate Gene Database (http://pdgd.njau.edu.cn:8080/) was used to query information about five replication modes [67]. At the same time, (a) sequence coverage length > 80%; (b) alignment region identification > 80% as the standard to identify duplicated genes [68]. In order to further explore the evolutionary pressure, DnaSP software was used to calculate the Ka, Ks and Ka/Ks values of the identified duplicated gene pairs [69]. The syntenic genes information of *B. rapa*, *B. oleracea*, *B. napus* and *A. thaliana* were queried in BRAD database [24], and the circos diagram was drawn using TBtools to express the syntenic relationships among *B. rapa*, *B. oleracea* and *B. napus* [70].

### Phylogenetic relationship analysis

We collected MBD protein sequences of four dicotyledons (*B. rapa*, *B. oleracea*, *B. napus* and *A. thaliana*) and two monocotyledons (*Oryza sativa* and *Zea mays*). Using ClustalX to perform multiple sequence alignments of their sequences, MEGA X used the alignment results to construct a Maximum Likelihood (ML) tree with 1000 bootstrap repeats [71]. The Interactive Tree of Life (https://itol.embl.de/) was the final beautification of the phylogenetic tree [72].

### Conserved motifs and MBD domains analysis

The 20 motifs of *B. napus* and its ancestors MBD proteins were searched on the online website The MEME Suite (https://meme-suite.org/meme/) [73]. Easy Sequencing in PostScript (ESPript) 3.0 (https://espript.ibcp.fr/ESPript/ESPript/index.php) [74] was used to draw the conserved domains map, which the sequence data of MBD proteins in *B. rapa*, *B. oleracea*, *B. napus* and *A. thaliana* were collected from Pfam database and compared by ClustalW.

### Gene Ontology enrichment analysis

To predict the potential functions of genes and understand biological pathways, TBtools was used to perform Gene Ontology (GO) enrichment analysis of *MBD* genes in *B. rapa*, *B. oleracea* and *B. napus* [70].

### Analysis of *MBD* genes expression in stems, leaves, flowers and siliques

*B. napus* (cv. Darmor) and its diploid ancestors, *B. rapa* (cv. Chiifu) and *B. oleracea* (cv. Jinzaosheng), were planted in natural environment in Wuhan, China, and the seeds came from the Oil Crops Research Institute, Chinese Academy of Agricultural Sciences. Plant materials were collected and studied with institutional permission and in accordance with national standards. The stems, leaves, flowers, and siliques (10 days after pollination) of healthy 6-month-old plants were collected and immediately frozen in liquid nitrogen. *MBD* gene expression patterns in four tissues were analyzed using Illumina RNA-seq data [75]. After normalization of FPKM values, HemI software was used to present heatmap [76].

## Supplementary Information

**Additional file 1: Table S1.** The five duplicated types of *MBD* genes in *B. napus* and its ancestors.

**Additional file 2: Table S2.** The upstream and downstream transposable elements of *MBD* genes.

**Additional file 3: Figure S1.** GO enrichment analysis of *B. rapa* (A), *B. oleracea* (B) and *B. napus* (C). Blue is molecular function, red is cellular component.

**Additional file 4: Table S3.** MBD family member characteristics and subcellular localization.

**Additional file 5: Table S4.** Synteny analysis of *MBD* genes in *B. rapa*, *B. oleracea*, *B. napus* and *A. thaliana*.

**Additional file 6: Table S5.** FPKM values of *MBD* genes in *B. rapa*, *B. oleracea* and *B. napus*.

**Additional file 7: Table S6.** The |log_2_FC| of *MBD* genes in four tissues.

## Data Availability

The datasets supporting the conclusions of this article are included within the article and its additional files. The raw RNA-seq data of the flowers, leaves, siliques and stems of *B. rapa*, *B. oleracea* and *B. napus* came from the NCBI database (https://www.ncbi.nlm.nih.gov//bioproject/489323), with the accession number (SRR7816633-SRR7816668). Public access to the database is open.

## Abbreviations

*AA*: *A. thaliana: Arabidopsis thaliana*
*B. rapa*: *Brassica rapa*
*B. oleracea*: *Brassica oleracea*
*B. napus*: *Brassica napus*
BLAST: Basic Local Alignment Search Tool
DSD: Dispersed duplication
CDS: Coding sequences
ESPript: Easy Sequencing in PostScript
FPKM: Fragments per kilobase million
GRAVY: Grand average of hydropathicity
GSDS: Gene Structure Display Server
ISWI: Imitation SWItch
Ka: Non-synonymous substitution
KDa: kilodaltons
Ks: Synonymous substitution
LF: Least fractionated blocks
MBD: Methyl-CpG-Binding Domain
MF1: Medium fractionated blocks
MF2: Most fractionated blocks
ML: Maximum Likelihood
MW: Molecular weight
PD: Proximal duplication
PI: Isoelectric point
PlantCARE: Plant *Cis*-Acting Regulatory Element
RU: Repbase Update
SRA: SET and RING finger-associated domain
TD: Tandem duplication
TRD: Transposed duplication
TSS: Transcription start site
WGD: Whole genome duplication
WGT: Whole-genome triplication
