# Supplementary figures and images for: The impacts of allopolyploidization on Methyl-CpG-Binding Domain (MBD) gene family in Brassica napus
Source: BMC Plant Biol. 2022 Mar 7;22:103. doi: 10.1186/s12870-022-03485-0 (PMC8900393; doi:10.1186/s12870-022-03485-0)

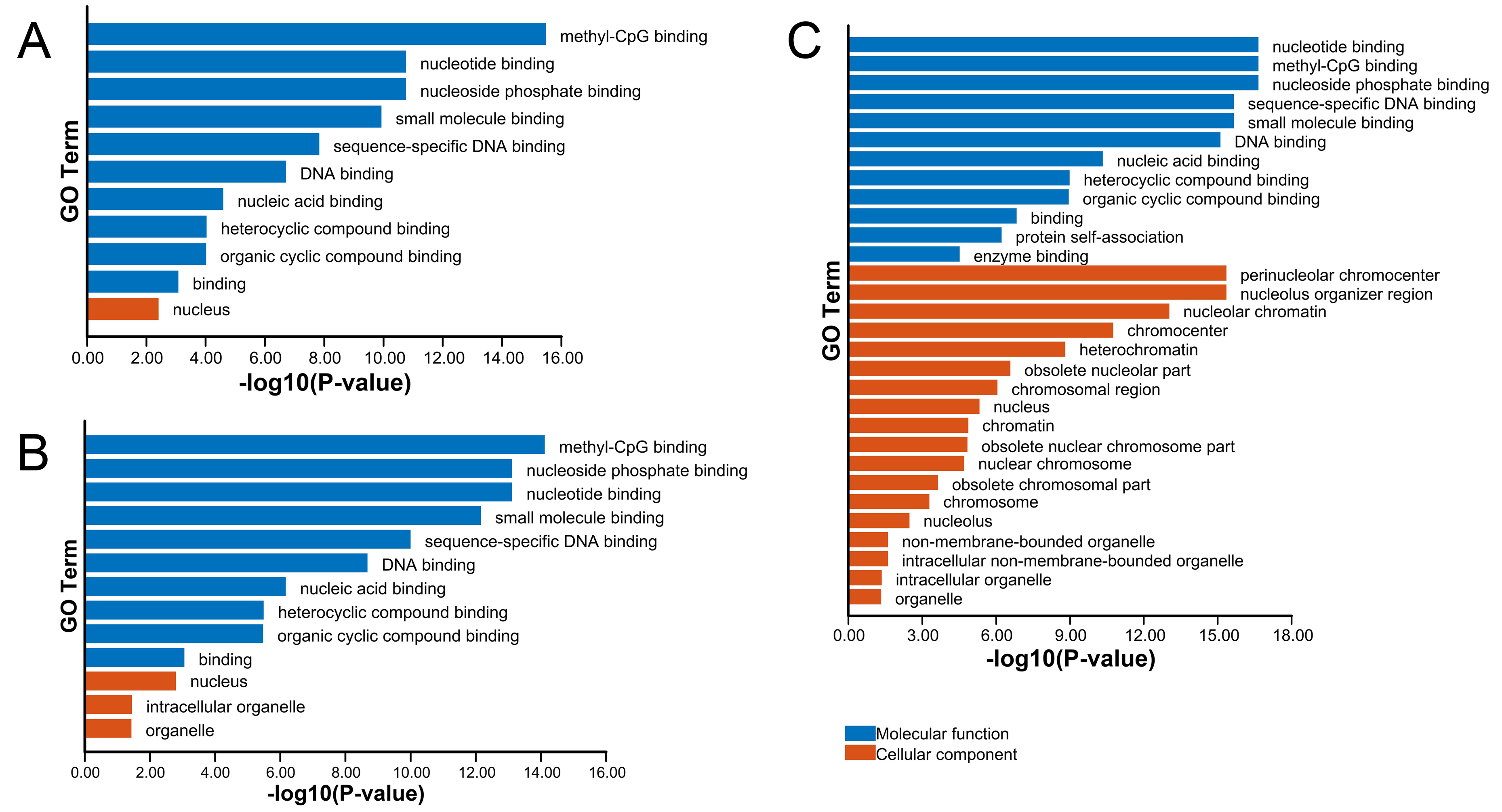

Supplement: Supplementary file 3 — Additional file 3: Figure S1. GO enrichment analysis of B. rapa (A), B. oleracea (B) and B. napus (C). Blue is molecular function, red is cellular component. [file 12870_2022_3485_MOESM3_ESM.tif]
